# ‘Pamoja Tunaweza’: A Collaborative Program Model for Global Health Training & Education

**DOI:** 10.5334/aogh.3791

**Published:** 2022-10-26

**Authors:** Jeanne Moseley, Innocent Mboya, Mia Haller, Emily Lasher, Caroline Amour, Sia Msuya, Rachel Manongi

**Affiliations:** 1 Global Health Program, Cornell University, US; 2 Institute of Public Health, Kilimanjaro Christian Medical University College, TZ; 3 Department of Community Health, Kilimanjaro Christian Medical Center, TZ

**Keywords:** global health, global health education, academic partnership

## Abstract

The past two decades have seen unprecedented student demand for global health education through experiential and engaged learning within institutions of higher education in the United States. This demand created a need for innovative institutional partnerships. Since 2007, faculty from Cornell University’s Global Health Program and Kilimanjaro Christian Medical University College (KCMUCo) have worked together to design, implement, and evaluate an innovative experiential learning program in global health and development policy. Since its inception, 176 Cornell undergraduates and 188 KCMUCo medical students have successfully engaged in the policy case study collaborative program and course, leading to the development of 75 policy case studies.

This long-term partnership between Cornell and KCMUCo has led to an innovative cross-cultural teaching model, funding support, professional presentations for students and faculty, a visiting scholars program at Cornell, and new avenues for research and collaboration. Fifteen years of sustained partnership has required the navigation of several unique and complex challenges, providing the opportunity to reimagine and strengthen this program and partnership. The objective of this article is to share a powerful program model for global health training and education, and discuss the challenges, successes, and lessons learned through this continued collaboration.

## Background

The past two decades have seen unprecedented student demand for global health education and engaged learning in institutions of higher education in the United States [1,2]. In response, in 2006, Cornell University applied for and received a Framework Programs in Global Health Grant from the National Institutes of Health, which sought to develop multidisciplinary global health programs that fostered opportunities for innovative curriculum development, global health training, and institutional collaborations. With this grant, Cornell University developed an undergraduate Global Health Program which has supported students pursuing global health education through a university-wide Global Health minor beginning in 2007 and a Global and Public Health Sciences major beginning in 2014.

With this increased demand for global health education came the need for innovative institutional partnerships [3]. In 2007, a Cornell University seed grant brought partners from Cornell University in Ithaca, New York and Kilimanjaro Christian Medical University College (KCMUCo) in Moshi, Tanzania together for exploratory partnership conversations. This led to the development and submission of an International Science and Education Grant through the United States Department of Agriculture. With this three-year grant award, Cornell and KCMUCo faculty worked together to design, implement, and evaluate an innovative experiential learning program in global health and development policy that engages equal numbers of Cornell and KCMUCo students. This initial funding allowed for the development of a long-term tuition-based funding model which has continued to financially sustain the program and partnership [4].

This program was founded on principles of educating and inspiring future global health leaders and practitioners from diverse backgrounds, going beyond didactic teaching and learning. The program was tailored to engage and expand students’ critical thinking capacities, interpersonal and cross-cultural skills, and frames of reference through a mutually rewarding partnership between low-income and high-income country institutions [1]. Between 2009 and 2022, 176 Cornell undergraduates and 188 KCMUCo medical students successfully engaged in the collaborative program and course, leading to the development of 75 policy case studies.

Recent literature and dialogue has called for an examination of global health, global health training programs and the inequities and power imbalances that are pervasive in global health work and partnership [3,5,6,7]. We write from the perspective of educators in the United States and Tanzania who have worked together for 15 years, consistently grappling with the challenges and inequities inherent in international institutional partnerships as we educate students who aspire to careers in global health. Since its inception, this program has been noted as an example of a collaborative and mutually beneficial partnership, one that we have worked diligently to build and sustain [4,8].

While there is considerable literature discussing global health research and training partnerships in clinical education settings, there is a need for further documentation of undergraduate and non-clinical collaborative global health program models [9,10]. The objective of this article is to share a powerful program model for global health training and education, and discuss the challenges, successes, and lessons learned through this continued collaboration.

## Methodology

### Course Structure Overview

In this collaborative program, students come together at KCMUCo in teams of five to six Cornell and KCMUCo students to develop, write, and present a new global health policy case study on a topic relevant to Tanzania. Through this process, groups work together to analyze a policy problem, identify policy issues and evaluate relevant policy options. This offers participants the opportunity to develop broad knowledge about global health and development issues in Tanzania. In addition to academic deliverables, students must demonstrate effective engagement in a cross-cultural learning environment through participation in reflective practices related to cultural diversity, professional growth, collaboration, and service learning. The following sections expand on who we bring together, the policy case study approach, and collaborative models for learning and support.

### Who We Bring Together

#### Institutional engagements

From the initial stages, this collaborative program has been developed and sustained through equitable long-term institutional commitments. Many partners at both KCMUCo and Cornell come together each year to prepare, develop, and offer this program. While students and faculty are crucial to the success of this collaboration, this partnership also depends on support from staff members in administrative, legal, and budget offices at both institutions. This requires cultivation of relationships across Cornell and KCMUCo in order to successfully execute contracts, legal agreements, budgets, and logistics. The program also relies on the support of affiliated faculty and staff to offer additional instruction that prepares students to engage in this program. For example, Kiswahili instructors and expert stakeholders teach students about health systems and critical public health issues in Tanzania.

#### Students

**Figure 1:**
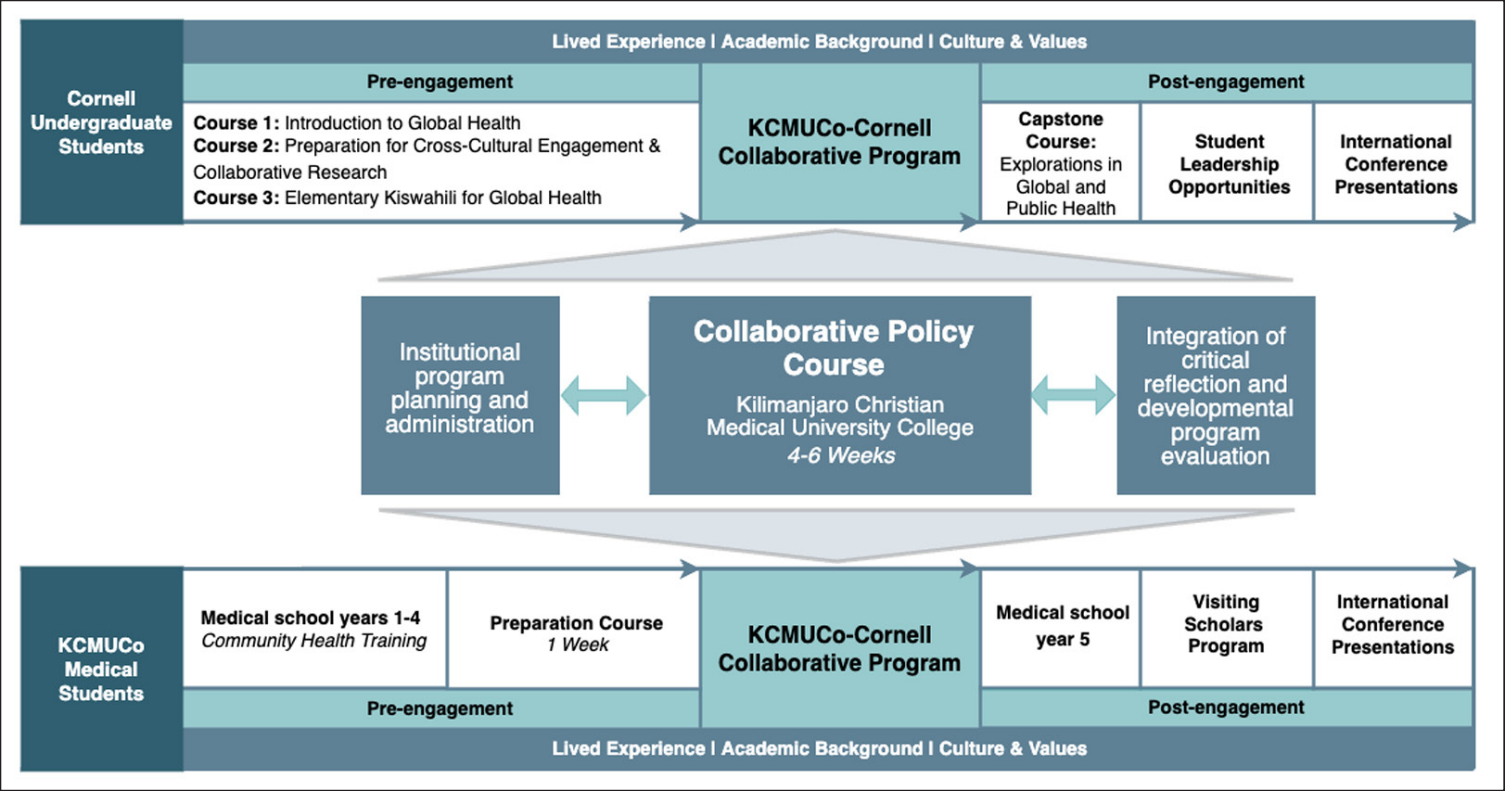
The Cornell-KCMUCo Global Health Collaborative Program Model.

This program brings together equal numbers of students from Cornell and KCMUCo in Moshi, Tanzania. Cornell undergraduates in the program are rising juniors or seniors, representing a diverse collection of academic majors and minors. KCMUCo students are fourth year undergraduate medical students in a five-year postsecondary program with interests in global and public health. Since the inception, KCMUCo and Cornell faculty have recognized the importance of a competitive application and rigorous preparation process for students at both institutions, acknowledging the unique academic knowledge, values, culture, and lived experience that each student brings to the program. The application process requires demonstration of academic excellence and commitment to engagement in the program. As demonstrated in Figure 1, Cornell students are required to complete three semester-long courses, *Introduction to Global Health, Preparation for Cross-Cultural Engagement and Collaborative Research, and Elementary Kiswahili for Global Health* prior to engaging in the program. KCMUCo students are required to complete a week-long preparation seminar where they are introduced to the policy case study analysis approach, literature search process, citation management strategies, writing skills, and elements of cross-cultural interaction. The preparatory courses are a critical opportunity for students to develop the skills necessary to engage successfully in this program and to demonstrate their preparedness and commitment to fulfilling their academic and professional responsibilities.

For both groups of students, the collaborative program is integrated into their coursework. For KCMUCo students, the program fulfills their research elective requirement. For Cornell students, the program serves as an Experiential Learning Opportunity, a requirement for students pursuing the Global Health minor or Global and Public Health Sciences major.

### Policy case study approach

A unique aspect of this program structure is the use of a policy case study approach as opposed to other documented frameworks [9,11]. KCMUCo and Cornell faculty work collaboratively to guide students in utilizing a policy case study approach to research an issue of public health concern in the Tanzanian context [12]. Students spend the first week of the course orienting themselves to the policy case study model and learning from guest experts about a wide variety of global health topics. This teaching pedagogy recognizes students as essential stakeholders and partners in global health dialogue. Problem identification is a collaborative effort between faculty and students with student groups receiving feedback from the teaching team and peers. Students work together to select a topic that is relevant to the Tanzanian context (Table 1). Students then conduct a review of both peer-reviewed and grey literature to understand the topic from a multidisciplinary perspective and inform the process of identifying expert stakeholders. After completing a literature search and gaining insights from stakeholder interviews, students identify policy issues and propose recommendations for policy options. The culmination of this policy research process is an extensive written policy case study and final presentation to faculty and community stakeholders.

**Table 1:** Examples of topic areas and titles of past case studies (2009–2022).


TOPIC AREA	TITLE	YEAR

**CommunicableDiseases**	Tuberculosis Infection Control in the Kilimanjaro Region: A Health Care System Perspective	2018

	Analysis of Acute Respiratory Infections in Children Under Five in Tanzania: A Policy Case Study	2019

	Antimicrobial Resistance: A Case Study in Tanzania	2022

**Environmental Health**	Unequal Access to Human Waste Management in Moshi Urban	2010

	Planetary Health: Energy Security and its Implications on Health Care Access, Delivery, and Quality in Tanzania	2019

	Impact of Outdoor Air Pollution on the Health of School-aged Children in Moshi Municipality	2022

**Health Systems**	Brain Drain of Health Professionals in Tanzania	2010

	Patient Waiting Times and its Effect on Healthcare Delivery in the Medical Outpatient Department of KCMC	2013

	Self-Medication Without Clinical Consultation in Urban Moshi, Tanzania	2018

**Injuries**	Increase in Motorcycle Accidents and Resulting Disability and Mortality in Moshi, Tanzania	2013

	Limited Emergency Medical Care Services in the Kilimanjaro Region	2022

**Maternal and Child Health**	A “Secret Disaster”: Intimate Partner Violence and Women’s Health in Moshi Urban and Rural	2016

	Barriers to Exclusive Breastfeeding Practices (0–6 months) Among Women in Moshi Urban and Rural	2016

	A Case Study Analysis of Perinatal Mortality in Tanzania	2019

**Mental Health**	Revealing the High Prevalence of Undiagnosed Mental Health Disorders in Urban Moshi, Tanzania	2015

	An Analysis of Poor Maternal Mental Health in Tanzania	2019

	Depression and Anxiety Among Medical Students in Tanzania and KCMUCo	2021

**Non-communicableDiseases**	Awareness of Non-communicable Diseases and Their Risk Factors Among Workers in the Urban Moshi, Tanzania Community	2014

	Prostate Cancer and the Burden of Disease in Tanzania	2015

	An Investigation of Type II Diabetes Among Adults in Urban Moshi	2019

**Nutrition**	Malnutrition in Children Residing in Orphan and Street Children Centers in Moshi, Tanzania	2013

	Aflatoxin Awareness: The Case Study of Moshi/Kilimanjaro	2015

	Undernutrition in Children Under 5 in Moshi Rural: A Health Systems Perspective	2016

**Sexual and Reproductive Health**	The Persistence of Unmet Need for Family Planning: The Disparity between Female Knowledge Holders and Male Decision Makers	2013

	Human Papillomavirus Vaccination Coverage in Tanzania	2015

	Social Vulnerability of Women Among Infertile Couples in Kilimanjaro Region, Tanzania	2018


### Collaborative Learning and Program Cornerstones

**Figure 2:**
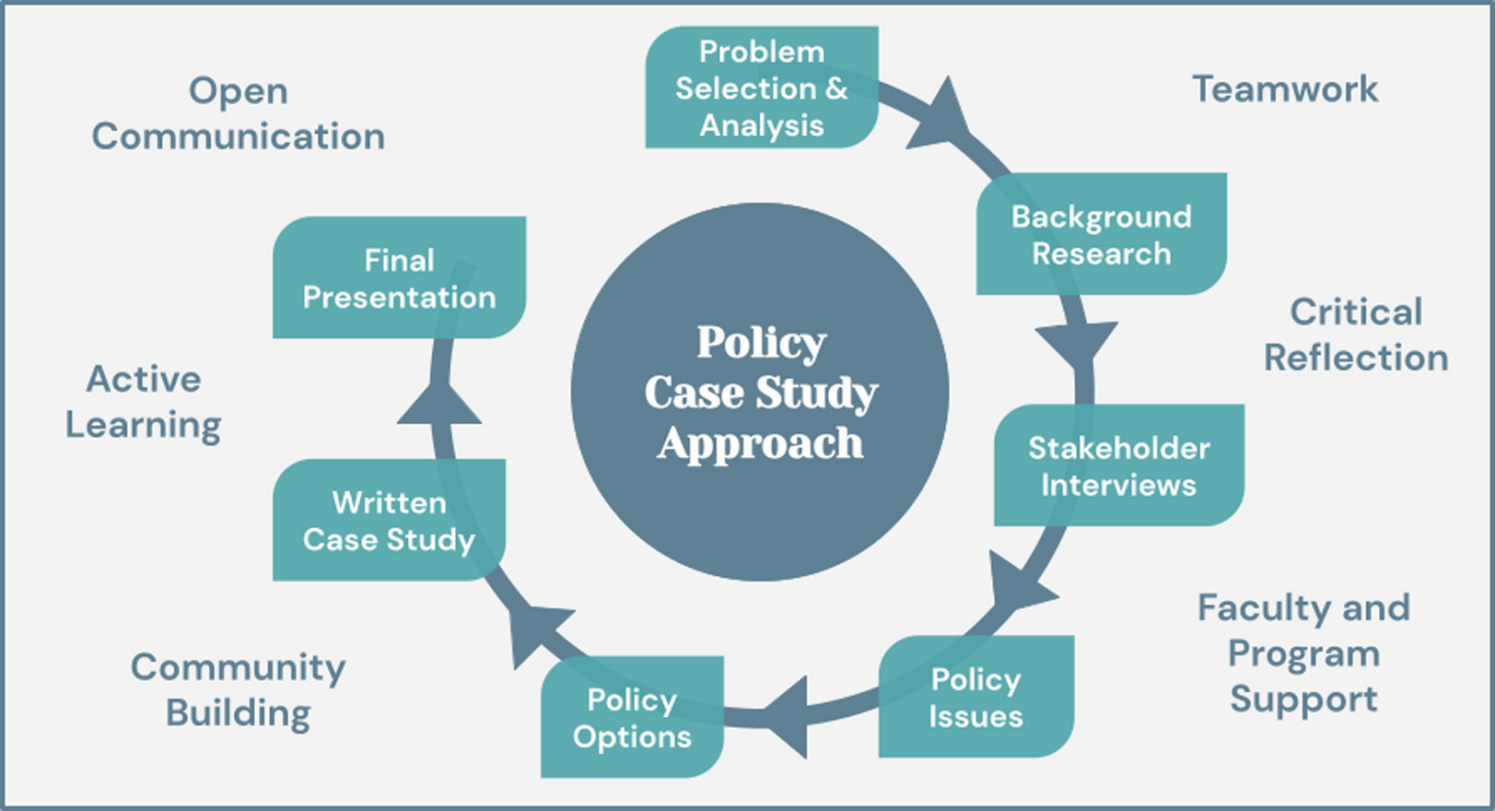
The program’s unique approach, combining a policy case study and critical global health skills and values.

In addition to the academic components of the policy case study structure, this model is guided by further pedagogical cornerstones including open communication, active learning, community building, teamwork, critical reflection, and faculty and program support (Figure 2). These values are intentionally and explicitly cultivated by the teaching team through facilitated group work, formal and informal discussions, establishing clear learning goals, direct feedback mechanisms, weekly critical reflection sessions and written prompts, and problem-based learning.

Faculty and program support involves mentorship, direct feedback, faculty presence during group work time, and facilitation of community building activities. The teaching team actively cultivates an environment where students feel valued and cared for which allows them to lean into the challenges of teamwork, ask for support, and learn from their mistakes.

Critical reflection serves as an example of a challenge, an element of support, and a pedagogical cornerstone in this program. Critical reflection is not only a foundational value, but also an invaluable tool of learning, which allows us to respond to diverse forms of dissonance. The program approaches critical reflection in two ways. First, through course-specific reflection related to content, mode of delivery, collaborative learning, newly acquired skills, and expectations for the next phase of training. This is essential to creating a harmonious learning environment. Second, interpersonal and cultural reflections bring in students’ own perspectives and lived experiences. This allows students to reflect on the personal identities that shape their participation in the program, allowing for mutual exchange of shared values and promoting a personalized sense of collaborative learning. Critical reflection is incorporated through weekly individual written assignments and group discussion activities tailored to encourage open mindedness, critical analysis of the learning process, and introspection. An integrated critical reflection practice creates opportunities for students to engage beyond the academic learning process.

## Challenges, Lessons Learned, Opportunities for Growth, and Achievements

Over the nearly 15 years of collaboration, faculty and students have had to navigate several unique and complex challenges to sustain this program and partnership. Through these challenges, we have learned many lessons about the principles and practices that are critical to continued partnership growth and advancement.

### Committing to reciprocity

Bringing together our two universities requires that all involved acknowledge and challenge the inherent inequities and power dynamics. This involves everything from aligning academic calendars to balancing institutional priorities and navigating different approaches to teaching and learning. A shared commitment to reciprocity through challenge is critical to sustaining and growing this partnership [7,13].

Since the inception of the program, faculty have always collaborated on program design, development and delivery of course content, assessment of student engagement, evaluation of program components, and investment in future partnership priorities and initiatives. The development of a visiting scholars program at Cornell is just one of the many outcomes of this commitment to reciprocity. Through the visiting scholars program, Cornell University hosts scholars from KCMUCo for a month each year to attend classes, explore academic interests, and interact with Global Health Program students in and out of the classroom. Reciprocity requires not only active collaboration, but also trusting relationships, open communication, and a genuine excitement and curiosity for the ways in which we can learn from each other. Faculty and partners strive to model for students that we can learn best by learning from one another in all aspects of program implementation.

### Fostering teamwork

We invite and expect all students to join the teaching team in reciprocal learning by engaging in deeply collaborative teamwork, which is time intensive, demanding, and often a challenge for students. It requires that all participants be open to navigating new forms of dissonance. Over the years, supporting students engaging in this type of personal and academic learning has required centering the value that we are co-creating a rich and unique opportunity to learn from one another across many forms of difference. This requires us all to lean into discomfort. Faculty and students must bring a sense of humility and cultivate relationships of mutual respect to not only fulfill academic responsibilities, but also learn from one another. While bringing together students and faculty across cultures, identities, and academic experiences can be a challenge, it is crucial to sustainably engaging in global health practice and training future global and public health professionals.

### Adapting to covid-19

With the disruption of in-person learning and international travel, the COVID-19 pandemic challenged the ways in which we were able to collaborate. The teaching team relied on shared values and past lessons learned as we took advantage of a unique opportunity to reimagine and strengthen this program and partnership utilizing virtual learning platforms [14].

Given the need to adapt the existing program format, faculty from Cornell and KCMUCo worked to generate a new instructional model for the 2021 program. Faculty members met weekly for nearly a year, reviewing and preparing not only curricular materials but also opportunities for community building and critical reflection. Faculty remained committed to creating a collaborative community across cultures and computer screens, and a meaningful engaged learning program for students. In doing this, we overcame the immense challenges inherent in this mode of instruction, including technological glitches, time zone differences, Zoom fatigue, and the complexities of building community in the virtual environment. Despite these barriers, the successful adaptation of this program demonstrated a shared commitment to this long-term partnership and its educational mission to train future global health leaders and collaborators. In 2022, the program was able to return to an in-person format with students and faculty coming together at KCMUCo in Moshi, Tanzania.

### Celebrating achievements

Over the years, 75 case studies have been successfully written and presented, with 12 student groups having their work accepted for presentation at international conferences.

Case studies have encompassed a wide range of topics and global health issues as depicted in Table 1.

In addition to the successes of the collaborative course, this partnership has created many additional opportunities for further research and collaboration at Cornell and KCMUCo. Faculty members from KCMUCo have been selected for participation in the WHO Cochrane Institute at Cornell. In addition, nineteen KCMUCo faculty members and eighteen KCMUCo medical students have traveled to Cornell as part of the visiting faculty and visiting scholars programs respectively. Many further collaborations have stemmed from this partnership, including a two-stage implementation research project titled, *Building Strong Nutrition Systems* in support of Scaling Up Nutrition in Tanzania. Additional collaborations include a Geographical Information Systems (GIS) workshop and short course at KCMUCo, a partnership between KCMUCo Institute of Public Health and Better Health for African Mother and Child (BHAMC), and the establishment of the Uzima Garden: Spaces of Nourishment and Healing Project.

## Conclusions

The achievements of this program model demonstrate that reciprocal and sustainable global health partnerships are built on 1) cultivating relationships grounded in mutual trust, respect, and open communication 2) addressing the inherent inequities between low-income and high-income institutions, and 3) creating transformative learning experiences for all students, faculty, and affiliated partners. Other institutions can learn from this model by evaluating how the presented programmatic components and partnership principles may be adapted and utilized towards navigating complex challenges and strengthening their own global health programs and collaborations.

## References

[1] Adams LV, Wagner CM, Nutt CT, Binagwaho A. The future of global health education: training for equity in global health. BMC Med Educ. 2016; 16(1): 296. DOI: 10.1186/s12909-016-0820-0 27871276 PMC5117699

[2] Mendes IAC, Ventura CAA, Queiroz AAFLN, Sousa ÁFL de. Global Health education programs in the Americas: A scoping review. Ann Glob Health. 2020; 86(1): 42. DOI: 10.5334/aogh.2745 32346523 PMC7181949

[3] Withers M, Press D, Wipfli H, et al. Training the next generation of global health experts: experiences and recommendations from Pacific Rim universities. Glob Health. 2016; 12(1): 34. DOI: 10.1186/s12992-016-0162-z PMC4918191 27334947

[4] Martin NA, Kalbarczyk A, Nagourney E, et al. Bending the arc towards equitable partnerships in global health and applied training. Ann Glob Health. 2019; 85(1): 130. DOI: 10.5334/aogh.2564 31750079 PMC6838765

[5] Macfarlane SB, Jacobs M, Kaaya EE. In the name of global health: trends in academic institutions. J Public Health Policy. 2008; 29(4): 383–401. DOI: 10.1057/jphp.2008.25 19079297

[6] Kreitlow A, Steffens S, Jablonka A, Kuhlmann E. Support for global health and pandemic preparedness in medical education in Germany: Students as change agents. Int J Health Plann Manage. 2021; 36(S1): 112–123. DOI: 10.1002/hpm.3143 33704858 PMC8207038

[7] Umoren RA, Einterz RM, Litzelman DK, Pettigrew RK, Ayaya SO, Liechty EA. Fostering reciprocity in global health partnerships through a structured, hands-on experience for visiting postgraduate medical trainees. J Grad Med Educ. 2014; 6(2): 320–325. DOI: 10.4300/JGME-D-13-00247.1 PMC4054735 24949140

[8] Crane J. Scrambling for Africa? Universities and global health. The Lancet. 2011; 377(9775): 1388–1390. DOI: 10.1016/S0140-6736(10)61920-4 21074254

[9] Liu Y, Zhang Y, Liu Z, Wang J. Gaps in studies of global health education: An empirical literature review. Glob Health Action. 2015; 8(1): 25709. DOI: 10.3402/gha.v8.25709 25906768 PMC4408318

[10] John CC, Ayodo G, Musoke P. Successful global health research partnerships: What makes them work? Am J Trop Med Hyg. 2016; 94(1): 5–7. DOI: 10.4269/ajtmh.15-0611 26483123 PMC4710444

[11] Rowson M, Smith A, Hughes R, et al. The evolution of global health teaching in undergraduate medical curricula. Glob Health. 2012; 8(1): 35. DOI: 10.1186/1744-8603-8-35 PMC3539925 23148763

[12] Pinstrup-Andersen P, Cheng F. Case Studies in Food Policy for Developing Countries: Policies for Health, Nutrition, Food Consumption, and Poverty. Cornell University Press; 2018.

[13] Citrin D, Mehanni S, Acharya B, et al. Power, potential, and pitfalls in global health academic partnerships: Review and reflections on an approach in Nepal. Glob Health Action. 2017; 10(1): 1367161. DOI: 10.1080/16549716.2017.1367161 28914185 PMC5645653

[14] Mirza A, Gang L, Chiu T. Utilizing Virtual exchange to sustain global health partnerships in medical education. Ann Glob Health. 87(1); 24. DOI: 10.5334/aogh.3179 33747799 PMC7954178

